# Evolution of nonenzymatic browning during the simulated Msalais‐production process in models of grape juice

**DOI:** 10.1002/fsn3.2829

**Published:** 2022-04-20

**Authors:** Rui‐li Zhang, Meng‐Meng Zhang, Yun‐Feng Pu, Li‐Xia Zhu

**Affiliations:** ^1^ Production and Construction Group Key Laboratory of High‐Quality Agricultural Product Extensive Processing in Southern Xinjiang Alar China; ^2^ 12483 College of Life Science Tarim University Alar China

**Keywords:** boiled grape juice, maillard reaction, model, msalais, nonenzymatic browning, wine

## Abstract

Msalais is a wine fermented from boiled grape juice. Nonenzymatic browning (NEB) greatly affects the quality of Msalais, but to date its mechanism has not been systematically analyzed. In the current study, the evolution of NEB during Msalais production was investigated using models of grape juice. 5‐Hydroxymethylfurfural (5‐HMF), browning index (BI), yellowness index (YI), sample absorbance at 420 (A420), and b* increased during heating, with a clear transition point at 110 min. The Maillard reaction (MR) was the major contributor to NEB. Vitamin C (VC) facilitated NEB in the late stage of heating. During heating that lasted over 130 min, glucose contributed to NEB more than fructose, while the reverse was true for heating lasting less than 130 min. Proline (Pro) was the most important amino acid in facilitating NEB. BI and A420 decreased during fermentation, while increasing slightly during wine storage. In conclusion, this study identified the evolution of NEB during the Msalais process, which will facilitate the control of traditional Msalais production for improved wine quality.

## Graphical abstract

5‐Hydroxymethylfurfural (5‐HMF), browning index (BI), yellowness index (YI), sample absorbance at 420 (A420), and b* increased during heating, with a clear transition point at 110 min. The Maillard reaction (MR) was the major contributor to nonenzymatic browning (NEB). Vitamin C (VC) facilitated NEB in the late stage of heating. During heating that lasted over 130 min, glucose contributed to NEB more than fructose, while the reverse was true for heating lasting less than 130 min. Proline (Pro) was the most important amino acid in facilitating NEB. BI and A420 decreased during fermentation, while increasing slightly during wine storage.

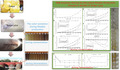

## 1 INTRODUCTION

Msalais is produced by a specific technique involving fermentation of a concentrated local grape juice in China. It is made by the Uyghur people in Xinjiang, northwest China, where the production technique has been inherited for thousands of years. Concentrated grape juice is used to naturally ferment into Msalais, and is responsible for the unique characteristics of Msalais, such as its typical brown color and strong caramel odor. These characteristics are due to nonenzymatic browning (NEB) that occurs during heating, and make Msalais distinct from regular wines.

Thermal processing improves food value attributes, such as organoleptic properties and, importantly, food health attributes (Dhungel et al., 2020; Hong et al., 2020; Parisi & Luo, 2018; Rufián‐Henares & Pastoriza, 2016). Heating of the food matrix results in a chain of chemical reactions, collectively known as NEB, giving rise to the formation of brown pigments and associated with the modification of the color, odor, and taste of the thermally treated foods (Hodge, 1953; Rufián‐Henares & Pastoriza, 2016). The effects of NEB are desirable in many foods, such as breakfast cereal, beer, and balsamic vinegar, in which the caramel aroma and color are expected (Choachamnan et al., 2020; Rufián‐Henares & Pastoriza, 2016; Tounsi et al., 2020; Van, 2006). Similarly, in fruit processing or cooking, NEB reactions play an important role by contributing to the flavor and aroma of food (Kader et al., 1997; Wu, 2014). Hence, the desirable effects of NEB accompanying grape juice concentration are important for the quality of Msalais. Conversely, the typical flavor and desirable brown color cannot be achieved if the grape juice is not sufficiently heated or, conversely, is overheated. Traditionally, the color of Msalais varies from light red to dark brown, and the aroma of Msalais is of a caramel odor (Zhu et al., 2013). However, how NEB evolves during the Msalais process has not, to date, been systematically analyzed.

NEB associated with the heating of the food matrix is attributed to several reactions, including the Maillard reaction (MR), caramelization, vitamin C (VC) browning, and pigment destruction (Hrynets et al., 2019; Kader et al., 1997; Rufián‐Henares & Pastoriza, 2016). Furthermore, polyphenols, fatty acids, and other carbonyl compounds can participate in the MR, even if formed via oxidative reactions (Bozkurt et al., 1999; Kader et al., 1997; Sajib & Undeland, 2020; Wang et al., 2021; Zamora & Hidalgo, 2005). In fact, molecules other than reducing sugars participate in the MR. This is particularly true for some lipid oxidation products, phenolic compounds, or any other molecules with carbonyl groups (Sajib & Undeland, 2020). MR and VC degradation, rather than caramelization, are reportedly the main NEB reactions during the concentration of grape juice (Bozkurt et al., 1999). In our previous work, the typical color and flavor of Msalais are formed as a result of NEB during heat concentration, and are due to MR, caramelization, and amino acid degradation (Meng‐meng et al., 2018; Zhang, 2016). Also, during fermentation and aging, polyphenol oxidation may be an important pathway for additional NEB (Zhang, 2016). Nevertheless, the available information on various NEB reactions in different food matrices or models under different heating conditions emphasizes the complexity of NEB in fruit juice processing, which has hindered the development of an effective solution to mitigate browning (Paravisini & Peterson, 2018). Thus, in this study, we focused on NEB evolution from the MR during the Msalais process in a model without polyphenols, as it is well understood that NEB caused by complex polyphenols is a complicated process.

Historically, the formation of brown color has been determined by using direct and indirect approaches. The former includes chemical methods for measuring the concentration of browning reaction products, such as 5‐hydroxymethylfurfural (5‐HMF) (Wang et al., 2006). 5‐HMF is an intermediate of brown pigment formation during the MR, and the level of 5‐HMF is strongly correlated with the degree of browning (Lee & Nagy, 1988; Wang et al., 2006). Conversely, indirect approaches focus on registering the variation of color associated with NEB by measuring sample absorbance at 420 nm (A420), a wavelength widely used for the detection of browning pigments (Ibarz et al., 2000). Another indirect technique is based on the CIE L*a*b* coordinates. Furthermore, BI associated with fruit has been extensively developed to evaluate both NEB and enzymatic browning (Karabagias et al., 2018; Maskan, 2001; Pathare & Opara, 2013).

Color resulting from the concentration of grape juice is the most important characteristic of Msalais quality. However, no systematic studies into Msalais color development have been reported to date. Accordingly, with the increase in Msalais consumption, oenologists are becoming increasingly interested in understanding the ways in which browning reactions can be controlled to achieve a beverage with improved organoleptic and health properties. The current study was designed to analyze the evolution of NEB during simulated Msalais processing. We analyzed multiple indicators, including L*a*b*, 5‐HMF, and A420, and used suitable model grape juice models to elucidate the processes that underpin color changes during Msalais production.

## 2 MATERIALS AND METHODS

### 2.1 Chemical reagents

Methanol, acetonitrile, 18‐amino acid mix, glucose, fructose, 5‐HMF, VC, and phenolic acids were all HPLC‐grade and purchased from Sigma‐Aldrich (USA). For the combined models in the study, individual amino acids (biological grade), phenolic acids (analytical grade), glucose (biological grade), fructose (biological grade), citric acid acids (biological grade), di‐potassium L(+)‐tartrate H_2_O (biological grade), DL‐malic acid (biological grade), VC (biological grade), and salts (analytical grade) were purchased from Shanghai Yuan Ye Biotechnology Co., Ltd. (China). Double‐distilled water was used in all experiments.

### 2.2 Models with different main compositions of grape juice

The ‘complete’ model was designed as the our previous formulation to simulate the grape juice of *Vitis vinifera* Hetianhong (Zhu et al., 2017). The other models were prepared by subtracting specific components from the ‘complete’ model, as specified in Table 1. The reagents were dissolved in 1 L of double‐distilled water. The specific quantities of the reagents in the complete and subtracted models are shown in Table 2. The pH of the models was determined using a pH meter (Sartorius AG, China) and adjusted to 3.5 with 1 M di‐potassium L(+)‐tartrate H_2_O. The models were heated with stirring (800 rpm) to bring to boil, to approximately 28 Brix, using a multihead temperature‐controlled magnetic stirrer. During heating, samples were collected from the initial model (time 0), at the time at which the model started to boil (approximately 30 min), and after the model had been boiling for 50, 70, 90, 110, 130, 150 170, and 190 min, respectively. To stop the browning reactions, the samples were immediately cooled under a stream of cold water. Vigorously fermenting cultures from a modern manufacturing plant (DaolangMslais Limited (Md)) and a traditional craft workshop (Abudu•GayitMsalais (Ma)) from the Awat region were used to inoculate the boiled ‘complete’ model at a 1% (v:v) ratio. The fermentation was then allowed to proceed at 25°C. Fermenting samples were collected on days 0, 7, 16, 25, 33, and 48, respectively. Each treatment was prepared in triplicate and samples were collected at the designated time points in triplicate.

**TABLE 1:** Compositions of the different model grape juice models

Model	Composition
Complete	Glucose, fructose, amino acids, phenolic acids, vitamin C (VC), salts
G + F + A	Glucose, fructose, amino acids
G + F + A + VC	Glucose, fructose, amino acids, VC
VC	VC
G + F	Glucose, fructose
A	Amino acids
P	Phenolic acids
Single amino	Individual amino acid, glucose, or fructose (24 models)

**TABLE 2:** The concentrations of the components in the grape juice models

Items	Compounds	Concentration
Sugars (g/L)	Glucose	110
	Fructose	110
Acids (g/L)	Citric acid	0.34
	Di‐Potassium L(+)‐tartrate H_2_O	5
	DL‐Malic acid	1.4
Amino acids (mg/L)	Proline (Pro)	569.36
	Tryptophan (Trp)	29.15
	γ‐Aminobutyric acid (GABA)	53.07
	Threonine (Thr)	34.67
	β‐Alanine (β‐Ala)	5.62
	Glycine (Gly)	4.82
	Arginine (Arg)	571.72
	Tyrosine (Tyr)	14.63
	Methionine (Met)	5.91
	Cystine (Cys)	13.3
	Leucine (Leu)	29.75
	Alanine (Ala)	59.1
	Valine (Val)	18.41
	Lysine (Lys)	37.41
	Histidine (His)	19.11
	Isoleucine (Ile)	12.39
	Asparagine (Asn)	12.78
	Serine (Ser)	18.18
	Glutamate (Glu)	46.43
	Ornithine (Orn)	17.18
	Phenylalanine (Phe)	31.76
	Aspartic acid (Asp)	22.31
	Glutamine (Gln)	34.08
Phenolic acids (mg/L)	Gallic acid	0.6
	Protocatechuic acid	3.25
	p‐Coumaric acid	0.35
	p‐Hydroxybenzoic acid	9.95
	Caffeic acid	1.14
	Ferulic acid	1.99
	Vanillic acid	6.97
	Clove acid	0.56
	Salicylic acid	7.14
Salts (mg/L)	MnSO_4_·H_2_O	0.16
	ZnSO_4_	0.16
	CoCl_2_•6H_2_O	0.8
	CuSO_4_.5H_2_O	0.04
	KI	0.04
	NaMoO_4_•2H_2_O	2
	Cacl_2_.6H_2_O	0.02
	H_3_BO_3_	0.04
	NaCl	200
Vitamin (mg/L)	Vitamin C (VC)	100

### 2.3 Determination of the CIE color coordinates and BI

The samples were analyzed using a Hunter Lab model D25 L optical sensor (Hunter Associates Laboratory, Inc., Reston, VA, USA). For the analysis, 60 ml of a sample was placed in a cylindrical optical cell. Reflectance values were obtained using a 45‐mm viewing aperture. The colorimeter had been operating for 1 h prior to use and was calibrated using black‐and‐white reference plates obtained from the manufacturer. The reported data are the average values of five measurements. Tristimulus color parameters L*, a*, and b* (Karabagias et al., 2018) were determined. The yellowness index (YI, indicating the degree of yellowness) (Rhim et al., 1999) and the browning index (BI) were calculated by using the following equations (Maskan, 2001):

$$\mathrm{BI}=100\times \left(\frac{X‐0.13}{0.17}\right)$$

$$\text{where}\;X=\frac{a^{*}+1.75L^{*}}{5.645+a^{*}‐3.013b^{*}}$$

$$\mathrm{YI}=\frac{142.86b^{*}}{L^{*}}$$

### 2.4 UV‐Visible (UV–Vis) absorption measurements

UV–Visible (UV–Vis) absorption values of the models were determined at 294 nm and 420 nm at room temperature (approximately 25°C) using a UV756 spectrophotometer (Shimadzu Co., Ltd., China). When necessary, appropriate sample dilutions were prepared with an optical density less than 1.5. Each measurement was performed in triplicate and the mean values were used to construct kinetic plots. The relative standard deviations of the triplicates were below 8.33%. Sample absorbance at 294 (A294) indicates the formation of intermediate compounds during the NEB reaction, while A420 corresponds to the accumulation of brown compounds. The polymerization degree of intermediate substances from NEB was calculated by dividing sample absorbance at 290 (A290) by sample absorbance at A420 (Barbanti et al., 1990).

### 2.5 HPLC analysis

Chromatographic analysis of 5‐HMF and reducing sugars was performed using a LC‐20AB Shimadzu Series HPLC (Shimadzu Technologies) equipped with a quaternary pump, an autosampler injection system, an Ultimate XB‐C 18 column (250 mm × 4.6 mm, 5 μm), a degasser, a photodiode array detector (PDA‐M20A) with UV/VIS detection set to 280 nm for 5‐HMF, and a differential detector (RID‐10A) for sugar analysis. The system was controlled using a Shimadzu ChemStation for Windows (Shimadzu Technologies). To detect 5‐HMF, HPLC analysis was conducted using a methanol/0.5% formic acid solution (v/v) as the mobile phase (with the following methanol flow gradient: 15/85–2 min, 50/50–24 min, 100/0–27 min, 100/0–29 min, and 15/85–33 min). The operating conditions were as follows: flow rate of 0.8 ml/min; 33‐min run; column temperature of 35°C; and injection volume of 10 μl. A calibration curve was prepared using an HMF standard (Sigma‐Aldrich). For reducing sugar detection, the mobile phase was 80% acetonitrile; isocratic elution at 40°C was used; the flow rate was set at 1 ml/min; the injection volume was 10 μl, and the measurement took 20 min. Each sample was injected in triplicate.

## 3 RESULTS

### 3.1 NEB analysis during simulated concentration of model grape juice

#### 3.1.1 NEB evolution during simulated concentration

**FIGURE 1:**
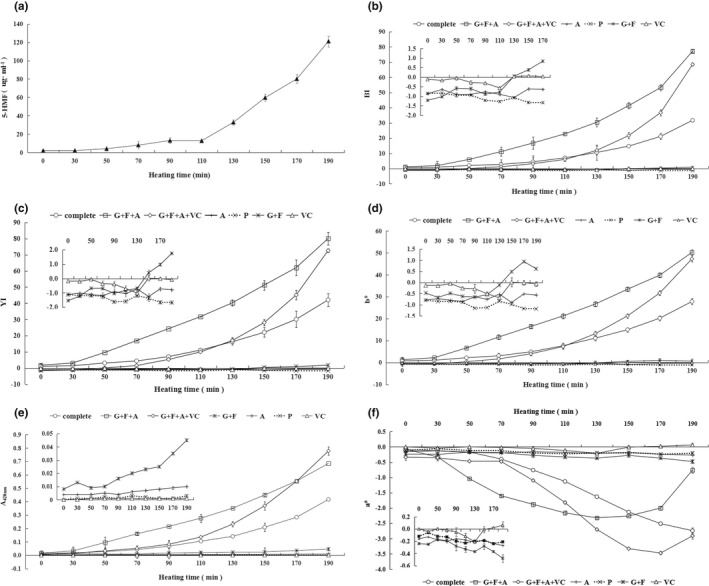
The effect of models with different compositions on the evolution of nonenzymatic browning (NEB) during the concentration. Changes in 5‐hydroxymethylfurfural (5‐HMF) levels during simulated concentration of the ‘complete’ model (a). Changes in the following were determined in seven models: Browning index (BI) (b), yellowness index (YI) (c), sample absorbance at 420 (A420) (d), a* (e), and b* (f). The insets in each panel show changes of BI (b), YI (c), A420 (d), a* (e), and b* (f) in the A, P, G + F, and VC models. The data are presented as the mean ± error from three independent experiments and three replicates

As shown in Figure 1a, the level of 5‐HMF increased in the ‘complete’ model with heating at the boiling temperature. There was a single, obvious transition point at 110 min. Before the 110‐min point, the level of 5‐HMF slowly increased, followed by a rapid linear increase after 110 min of boiling. The evolution curves of BI, YI, b*, and A420 (Figure 1b–e) were similar to that of 5‐HMF (Figure 1a). Namely, NEB increased with heating time, which was accompanied by an increase of BI, YI, A420, b*, and the level of 5‐HMF. Simultaneously, the a* value showed a downward trend during heating (Figure 1f).

Different model compositions impacted the evolution of NEB differently. Comparing the NEB rate, the first important models to consider are G + F + A, G + F + A + VC, and ‘complete’. As shown in Figure 1b–d, BI, YI, and b* increased rapidly in the G + F + A model, followed by the G + F + A + VC, and the ‘complete’ model. The observed increase in these indicators of NEB accelerated after 110 min, as noted for G + F + A + VC (Figure 1b–e). This indicated that NEB was slightly inhibited during the early heating period and then accelerated during the late heating period. For the G + F + A + VC model, the obvious increase of NEB after 110 min was mostly likely due to VC degradation, which then participated in and stimulated the NEB reaction.

Apart from sugars, amino acids, and VC, the ‘complete’ model contained phenolic acids and salts. NEB in the ‘complete’ model was not as pronounced as in the G + F + A and G + F + A + VC models, suggesting that phenolic acids and/or salts inhibited the reaction (Figure 1 b‐f). In other models containing only one sugar (G + F model), amino acids (A model), VC model, or phenolic acids (P model), NEB was much less pronounced than in the three models of G + F + A, G + F + A + V, and ‘complete’ (Figure 1b–f). NEB in G + F and VC models was slightly more pronounced than that in A and P models (Figure 1b–f). These findings suggest that in model grape juice, the MR of amino acids and sugars was the dominant contributor to NEB and that caramelization of sugars and VC degradation only slightly contributed to NEB.

#### 3.1.2 Contribution of reducing sugars and amino acids to NEB

**FIGURE 2:**
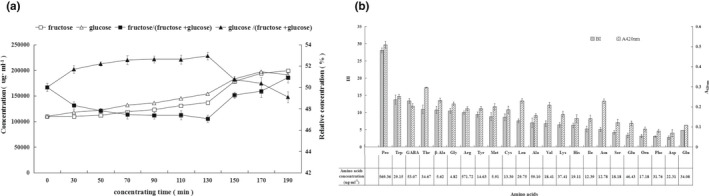
The trend of changing fructose and glucose levels during concentration of the ‘complete’ model (a), and the degree of NEB in different models of individual amino acids and reducing sugars (b)

As shown in Figure 2a, the concentration of glucose and fructose in the ‘complete’ model increased with time. Glucose levels were higher than fructose levels when the model was boiled for up to 150 min. The difference between glucose and fructose content decreased after 150 min, with the level of fructose exceeding that of glucose at 190 min. When the model was heated for less than 130 min, the relative concentration (percentage of the sum of fructose and glucose) of fructose decreased, while the relative concentration of glucose increased. However, when the model was heated for more than 130 min, the relative concentration of fructose increased and the relative concentration of glucose decreased. This suggests a somewhat higher consumption of fructose than glucose by the NEB reactions during heating before 130 min, and a much greater consumption of glucose than fructose after heating for more than 130 min, especially in the late stages of heating.

The degree of NEB in models containing individual amino acids and reducing sugars is shown in Figure 2b. In these models, the initial concentration of amino acids differed according to the composition of the natural grape juice (Figure 2b). The proline (Pro) model exhibited the highest NEB (BI ≥ 25, A420 > 0.4). The second highest NEB (25 > BI ≥ 10) was observed in the individual models of tryptophan (Trp), γ‐aminobutyric acid (GABA), threonine (Thr), arginine (Arg), glycine (Gly), β‐alanine (β‐Ala), and tyrosine (Tyr), respectively. In most models, A420 values were consistent with BI values: A420 > 0.3 was only observed in a model of proline, which was also characterized by the highest BI. The A420 values of models of asparagine (Asn), leucine (Leu), threonine, β‐alanine, tryptophan, glycine, methionine (Met), and valine (Val) were between 0.2 and 0.3. Hence, proline facilitated NEB to the greatest degree (BI > 25 and A420 > 0.3).

### 3.2 NEB during fermentation in a concentrated ‘complete’ model

**FIGURE 3:**
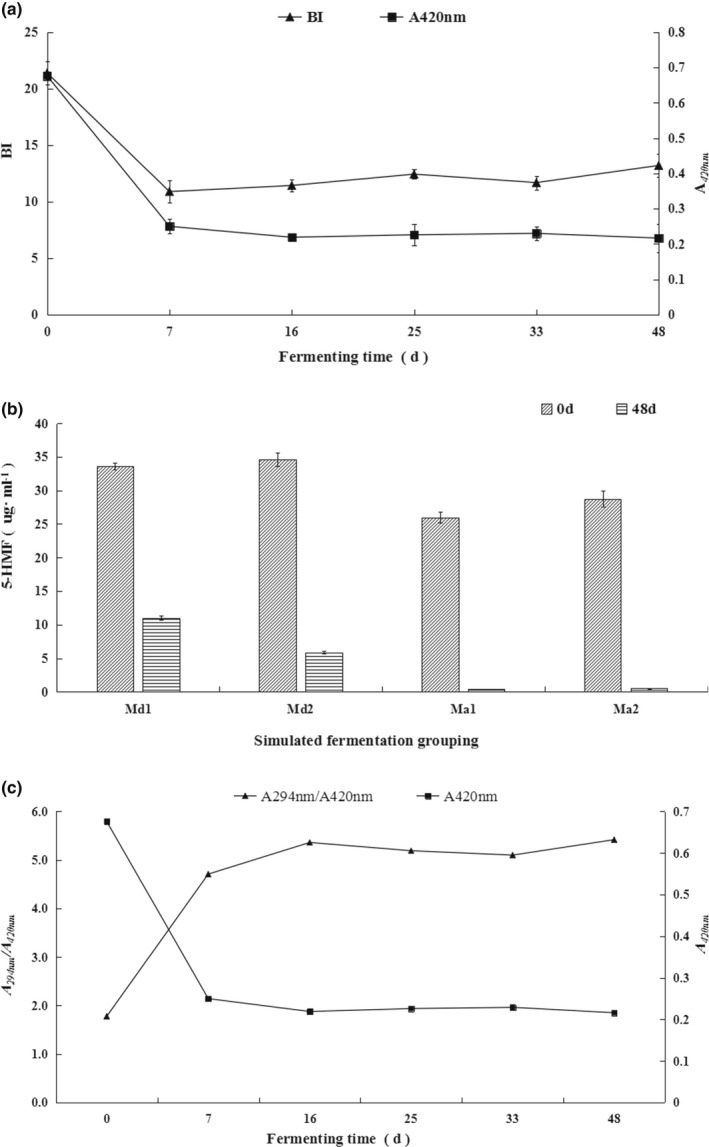
Nonenzymatic browning (NEB) evolution during fermentation of the concentrated ‘complete’ model. (a) Browning index (BI) and sample absorbance at 420 (A420) evolution; (b) changes in 5‐hydroxymethylfurfural (5‐HMF) evolution, fermentation after inoculation with vigorously fermenting cultures from a modern production plant (DaolangMslais Limited (Md)) and a traditional craft workshop (Abudu•GayitMsalais (Ma)); and (c) NEB polymerization degree (sample absorbance at 290 (A290)/A420)

BI and A420 decreased rapidly during the first 7 days of fermentation, from 21 to 10.8, and 0.65 to 0.24, respectively. Then, BI slowly increased to 13.5, while A420 slowly decreased to 0.21 (Figure 3a). Even though BI and A420 values decreased during fermentation, the level of 5‐HMF decreased rapidly in the four different concentrations of the ‘complete’ model (Figure 3b), with an increase in the polymerization degree of intermediate substances (A290/A420) produced by NEB during fermentation (Figure 3c). Meanwhile, BI increased slightly in the later stage of fermentation (Figure 3a).

### 3.3 Discussion

NEB contributes to the desired aroma, taste, and color of food to meet the expectations of consumers (Ismarti et al., 2021). In addition to this, the biological activities of some NEB products generated during the heating of food positively impact human health (Arihara et al., 2019; Dhungel et al., 2020; Nowak et al., 2021). Yet, on the other hand, the toxicity and undesired health effects of NEB products have been reported (Li et al., 2021; Nowak et al., 2021; Parisi & Luo, 2018). Currently, controlling NEB is critical for maximizing profit and avoiding formation of harmful compounds during food processing (Nowak et al., 2021; Parisi & Luo, 2018). During Msalais processing, regulating NEB is essential to take advantage of the MR to improve the sensory characteristics and to avoid harmful effects, such as eliminating or reducing the level of 5‐HMF. The data presented in this study describe the specific evolution of NEB in a model of heated grape juice, and form a firm basis for controlling, and thereby improving the traditional technique of Msalais production.

In the current study, it is well established that NEB increases with heating time of the food matrix. The level of 5‐HMF, BI, YI, A420, and b* all increased during heating of the ‘complete’ model, with one obvious transition point at 110 min. On the other hand, the a* value displayed a downward trend during the concentration stage. These observations agree well with the reported phenomenon of NEB, that is, a change of color, shifting from red to yellow and brown (Pathare & Opara, 2013). Besides the heating time, NEB was affected by the specific matrix components. The MR was the major contributor to NEB. In fact, the MR effectively takes place in boiling grape juice at temperatures >50°C and at a pH range of approximately 4–7 (Ajandouz et al., 2001), and NEB is still increasing in a concentrated grape juice at a very low temperature such as 5°C (Bakeshlou et al., 2020). VC is easily oxidized and decomposed through aerobic decomposition and anoxic decomposition to form dehydroascorbic acid, and then dehydrated to produce reductive ketones. These reductive ketones participate in the intermediate and final stages of the MR (Hrynets et al., 2019). In the current study, VC facilitated the MR during the late stage of heating instead of during early stage of heating, and this may be the high temperature to inhibit pathways of oxidative decomposition of VC in a degree (Wang et al., 2021). There are few reports on caramelization that occurred during the heating of juice or jam. That is because the boiling point of grape juice is far lower than the sugar melting point (>120°C) under acidic (pH 3) or alkaline (pH 9) conditions (Hrynets et al., 2019). In the current study, phenolic acids and/or salts inhibited the NEB reaction in boiling models (Figure 1b–f). In boiled grape juice, fructose is more reactive than glucose with respect to both 5‐HMF accumulation and NEB, during heating for 10 days at 55, 65, and 75°Cat pH 3.5 (Göğüş et al., 1998). Similarly, in sugar‐catalyst systems, fructose is the major reactant, leading to a more rapid formation of 5‐HMF than sucrose and glucose at pH 3.5 (Lee & Nagy, 1990). We showed here that the heating time is another important factor of NEB evolution. Fructose participated more actively in NEB evolution than glucose when model grape juice was heated for less than 130 min. However, glucose contributed to NEB more than fructose when heating lasted for more than 130 min.

The effect of amino acids in the model on NEB has often been investigated under the conditions of the same food matrix or food models (Hrynets et al., 2019). For example, under the same conditions (including concentration) glutamine (Gln) was more reactive than arginine with fructose as a substrate, while arginine was more reactive than glutamine with glucose as a substrate (Göğüş et al., 1998). According to the reaction rate of NEB, β‐alanine and tryptophan have been reported to be the most important amino acids for facilitating NEB due to their ability to enhance browning via the MR (Ajandouz & Puigserver, 1999). In contrast to these findings, we observed that the higher the amino acid content, the higher the degree of NEB, with proline being the most important amino acid contributor to NEB, possibly due to its high content in the models.

The other grape components in grape juice, such as polyphenol, are important for the color of regular wine, mainly due to anthocyanidins and flavonols (Gutiérrez‐Escobar et al., 2021). These factors should not be ignored for the NEB of Msalais, more possible with different pathways. According to the local oenologists of Msalais, the color of concentrated grape juice after the addition of grape residue extract is redder than that of the concentrated grape juice alone. This could be explained by the fact that polyphenol substances are extracted from grape skin and seeds. In the wine process, the substances including polyphenol in skins are often extracted during grape must fermentation, heating grape must before fermentation is sometimes used to increase the extraction and stabilization of wine color (Lisov et al., 2021). However, for Msalais, we have previously shown that the strong NEB occurred during the concentration of grape juice, although the total polyphenol content increases considerably, from less than 400 mg/L in grape juice to approximately 1300 mg/L in the final grape juice concentrate (Zhang, 2016). The NEB directly results in the deep brown color of Msalais wine (Zhang, 2016), instead of the red color or yellow color such as the red or yellow color in wine, attributed more to the anthocyanidins and flavonols (Gutiérrez‐Escobar et al., 2021). The increasingly concentrated polyphenol may be directly incorporated into MR, as nonenzymatic transglycosylation reactions, the main mechanism of phenolics’ incorporation into melanoidins (Moreira et al., 2017). Besides, the more concentrated the juice, the higher the anthocyanin and phenolic compound degradation (Mirzaee et al., 2016), these degraded compounds could also be incorporated into MR. In addition to these prior studies, our enhanced understanding of the evolution of NEB by the MR during the Msalais process provides a clear premise to further investigate the extent to which polyphenols contribute to NEB in Msalais. Additionally, the interaction between polyphenols and sugars during Msalais production, and its effect on color constitute an additional important research question.

## 4 CONCLUSIONS

The MR was the main contributor to NEB during Msalais production. 5‐Hydroxymethylfurfural (5‐HMF), browning index (BI), yellowness index (YI), A420, and b* increased during heating, with a clear transition point at 110 min. The Maillard reaction (MR) was the major contributor to NEB. Vitamin C (VC) facilitated NEB in the late stage of heating. During heating that lasted over 130 min, glucose contributed to NEB more than fructose, while the reverse was true for heating lasting less than 130 min. Proline was the most important amino acid in facilitating NEB. BI and A420 decreased during fermentation, while increasing slightly during wine storage. All in all, reduced sugars, amino acids (with proline as the most important amino acid), heating time, fermentation, and storage greatly impacted the degree of NEB in the final Msalais wine. These factors should be controlled to improve the color and health qualities of Msalais.

## CONFLICT OF INTEREST

The funders had no role in the design of the study; in the collection, analyses, or interpretation of data; in the writing of the manuscript or in the decision to publish the results.
